# Sequential intensified conditioning followed by prophylactic DLI could reduce relapse of refractory acute leukemia after allo-HSCT

**DOI:** 10.18632/oncotarget.8691

**Published:** 2016-04-11

**Authors:** Li Xuan, Zhiping Fan, Yu Zhang, Hongsheng Zhou, Fen Huang, Min Dai, Danian Nie, Dongjun Lin, Na Xu, Xutao Guo, Qianli Jiang, Jing Sun, Yang Xiao, Qifa Liu

**Affiliations:** ^1^ Department of Hematology, Nanfang Hospital, Southern Medical University, Guangzhou 510515, China; ^2^ Department of Hematology, Sun Yat-Sen Memorial Hospital, Sun Yat-Sen University, Guangzhou 510120, China; ^3^ Department of Hematology, the Third Affiliated Hospital, Sun Yat-Sen University, Guangzhou 510630, China; ^4^ Department of Hematology, Guangzhou General Hospital of Guangzhou Military Command, Guangzhou 510010, China

**Keywords:** donor lymphocyte infusion, refractory advanced acute leukemia, relapse, allogeneic hematopoietic stem cell transplantation, sequential intensified conditioning

## Abstract

The major obstacle is leukemia relapse for refractory leukemia undergoing allogeneic hematopoietic stem cell transplantation (allo-HSCT). We previously introduced a strategy of sequential intensified conditioning and early rapid immunosupressant withdrawal for refractory leukemia undergoing allo-HSCT, with 5-year overall survival (OS) and 3-year relapse rate of 44.6% and 33.3%. To reduce leukemia relapse, prophylactic donor lymphocyte infusion (DLI) was administered based on our historical strategy. A total of 153 refractory advanced acute leukemia patients were enrolled in this prospective study. According to the availability of donor lymphocytes and the criteria for DLI, 144 patients surviving day +60 were divided into two groups (80 DLI versus 64 non-DLI). The relapse rate was less and OS was better in patients receiving DLI than in those not receiving DLI (22.7% vs 33.9%, *P*=0.048; 58.1% vs 54.9%, *P*=0.043). The non-relapse mortality (NRM) was similar between DLI and non-DLI groups (*P*=0.104). Overall, the 5-year overall and disease-free survival post-transplantation were 51.1%±5.7% and 49.2%±5.3%. The 5-year relapse rate and NRM were 27.3%±4.4% and 29.7%±5.3%. Multivariate analysis revealed that lower bone marrow blasts on day 0, DLI and chronic graft-versus-host disease were associated with less relapse and better OS. The strategy of sequential intensified conditioning followed by early immunosupressant withdrawal and DLI could reduce relapse of refractory acute leukemia after allo-HSCT and improve survival.

## INTRODUCTION

Allogeneic hematopoietic stem cell transplantation (allo-HSCT) is commonly perceived as the only curable option for refractory leukemia [1-4]. However, patients with advanced stage who proceed directly to HSCT, especially those with high leukemia burden pre-transplantation, are likely to do poorly [5, 6]. The relapse rate exceeds 50% in these patients with standard myeloablative regimen consisting of total body irradiation (TBI) or busulfan (Bu) combined with cyclophosphamide (CY) [6, 7]. Some data showed that intensified conditioning could reduce leukemia relapse, but it might simultaneously increase non-relapse mortality (NRM) [6, 8, 9]. Therefore, maintaining the balance of efficacy and toxicity of conditioning is essential for long-term survival of recipient [10-12]. Recent studies have demonstrated that salvage chemotherapy with sequential conditioning could reduce leukemia relapse and had an acceptable toxicity for refractory leukemia [11-14].

In addition to conditioning regimens, the efficacy of allo-HSCT also relies on the graft-versus-leukemia (GVL) effect. We previously introduced a strategy of sequential intensified conditioning, consisting of fludarabine (Flu)/cytarabine (Ara-C) salvage chemotherapy followed by TBI/CY/etoposide (VP-16) myeloablative conditioning, and early rapid tapering of immunosuppressant for inducing GVL in refractory advanced leukemia undergoing allo-HSCT [15]. The results indicated that this strategy had an acceptable toxicity profile and improved outcome for refractory leukemia, with 5-year overall survival (OS) and disease-free survival (DFS) of 44.6% and 38.2% [15]. Despite these encouraging results, relapse in our cohort was still considerable, with 3-year relapse rate of 33.3% [15]. Based on these, in this study, we modified our historical strategy mainly by interventions with prophylactic donor lymphocyte infusion (DLI) for inducing GVL. The aim of this study was to evaluate whether this new strategy could reduce leukemia relapse and not increase NRM in refractory advanced acute leukemia.

## RESULTS

### Patient and transplant characteristics

Patient and transplant characteristics are summarized in Table 1. From January 2009 to June 2014, 153 patients with refractory advanced acute leukemia undergoing allo-HSCT were enrolled in the study group. Except for two who died of infections and two who died of regimen-related toxicities (RRTs) within 2 weeks post-transplantation, 149 patients surviving more than 30 days were evaluable for engraftment, disease response and Cyclosporine A (CsA) withdrawal.

**Table 1:** Patient, donor and transplant characteristics

Patient characteristics	Study group (n=153)	Historical group (n=48)	P-value
Female/Male	51(33.3%)/102(66.7%)	17(35.4%)/31(64.6%)	P=0.790
Median age, years (range)	29(12-57)	29(14-53)	P=0.837
Disease category			
AML	57(37.3%)	23(47.9%)	P=0.112
ALL	77(50.3%)	16(33.3%)	
ABL	19(12.4%)	9(18.8%)	
Genetics ^a^			
Favorable	3(2.0%)	4(8.3%)	*P=0.003
Intermediate	57(37.3%)	18(37.5%)	
Unfavorable	84(54.9%)	17(35.4%)	
Unknown	9(5.9%)	9(18.8%)	
Stage of treatment before transplantation			P=0.058
Primary induction failure	85(55.6%)	27(56.25%)	
Refractory relapse after CR1	46(30.1%)	15(31.25%)	
Refractory relapse after CR2	21(13.7%)	3(6.25%)	
Previous autologous transplantation	1(0.7%)	3(6.25%)	
Median BM blasts before conditioning (range)	30.0% (9.0%-96.0%)	24.0% (8.0%-92.0%)	P=0.436
Median circulating blasts before conditioning (range)	11.0% (0.0%-90.0%)	11.0% (0.0%-82.0%)	P=0.699
Median number of chemotherapy cycles before transplantation (range)	4 (2-8)	5 (2-13)	P=0.710
Extramedullary disease at the time of transplantation	16 (10.5%)	6 (12.5%)	P=0.693
CNSL	8 (5.2%)	2 (4.2%)	
Soft tissue and lymph node involvement	8 (5.2%)	4 (8.3%)	
Donor source			
Sibling donor	84(54.9%)	27(56.3%)	*P=0.018
Family donor	41(26.8%)	5(10.4%)	
Unrelated donor	28(18.3%)	16(33.3%)	
HLA typing			
HLA-identical	87(56.9%)	32(66.7%)	*P=0.003
One allele mismatched	12(7.8%)	8(16.65%)	
Two alleles mismatched	14(9.2%)	8(16.65%)	
Three alleles mismatched	12(7.8%)	0(0.0%)	
Four alleles mismatched	9(5.9%)	0(0.0%)	
Five alleles mismatched	19(12.4%)	0(0.0%)	
Stem cell source			
PBSCs	97(63.4%)	33(68.75%)	*P<0.001
BM	0(0.0%)	8(16.65%)	
PBSCs + BM	56(36.6%)	7(14.6%)	
Median CD34^+^ cells per graft, ×10^6^/kg (range)	9.1(5.4-12.7)	8.3(4.0-21.4)	P=0.681

AML=acute myelogenous leukemia; ALL=acute lymphoblastic leukemia; ABL= acute biphenotypic leukemia; CR1=first complete remission; CR2=second complete remission; BM=bone marrow; CNSL= central nervous system leukemia; PBSCs= peripheral blood stem cells; BM=bone marrow.

a genetics in the study group indicated cytogenetics and molecular genetics; genetics in the historical group only indicated cytogenetics.

* *P*<0.05.

Within the study population, 21 had comorbidities; 6 had hypertension, 3 had frequent ventricular premature beats, 8 had diabetes mellitus, and 4 had hypertension and diabetes mellitus. Two had a history of pulmonary tuberculosis, and 43 had a history of invasive fungal disease (IFD) pre-transplantation, including 19 with active Aspergillus pneumonia at the time of transplantation.

### Engraftment and disease response

All patients achieved hematopoietic reconstitution except for two who died of infections and two who died of RRTs within 2 weeks post-transplantation. The median time to neutrophil and platelet reconstitution were 12.0 (range, 9.0 to 31.0) days and 19.0 (range, 9.0 to 70.0) days, respectively. At the time of neutrophil reconstitution, 149 evaluable patients all achieved donor chimerism, including 61 with complete chimerism and 88 with mixed chimerism (with donor chimerism ranging from 67.0% to 95.0%). All 149 evaluable patients had complete chimerism by day +30 post-transplantation. At the time of neutrophil reconstitution, all 149 evaluable patients achieved complete remission (CR).

### RRTs

The conditioning was tolerated by all patients except two who died of RRTs. All 153 patients developed RRTs, and 44 grade III-IV RRTs. The incidence and mortality of RRTs were 100.0% and 1.3%. Toxicity was most common in the gastrointestinal tract. Organ toxicity is summarized in Table 2.

**Table 2:** Organ toxicity according to Bearman's criteria

	Grade I-II RRTs, n (%)	Grade III-IV RRTs, n (%)	Overall RRTs, n (%)
Heart	43(28.1)	9(5.9)	52(34.0)
Bladder	23(15.0)	16(10.5)	39(25.5)
Kidneys	11(7.2)	3(2.0)	14(9.2)
Lungs	4(2.6)	1(0.7)	5(3.3)
Liver	26(17.0)	10(6.5)	36(23.5)
CNS	9(5.9)	1(0.7)	10(6.5)
Mucosa	67(43.8)	23(15.0)	90(58.8)
Gut	104(68.0)	25(16.3)	129(84.3)

RRTs= regimen-related toxicities, CNS=central nervous system.

### Infections

Within the first 100 days post-transplantation, 85 patients developed 101 episodes of infections, including 33 bacteria, 12 fungi, 15 viruses (excluding viremia), 16 mixed infections (10 bacteria and fungi; 6 bacteria and viruses) and 9 infections of unknown etiology. Seven patients died of infections within the first 100 days post-transplantation. Active Aspergillus pneumonia was effectively controlled post-transplantation in the 19 patients with active IFD pre-transplantation. Compared with 257 acute leukemia undergoing allo-HSCT with standard myeloablative conditioning (TBI+CY, Bu+CY or Bu+Flu) at Nanfang Hospital at the same time, the incidence and mortality of infectious diseases within 100 days post-transplantation were similar between the two groups (*P*=0.296, *P*=0.730).

The 1-year cumulative incidences of cytomegalovirus (CMV)-emia, CMV-associated diseases, Epstein-Barr virus (EBV)-emia and EBV-associated diseases were 40.5%±4.1%, 4.2%±2.1%, 36.4%±5.5% and 18.7%±4.1%, respectively. The 1-year cumulative mortalities of CMV-associated diseases and EBV-associated diseases were 2.7%±1.9% and 5.9%±2.5%, respectively. Compared with 257 acute leukemia undergoing allo-HSCT with standard conditioning at Nanfang Hospital at the same time, the incidence of EBV-emia was higher in the study group (*P*=0.037), and the incidences of EBV-associated diseases, CMV-emia and CMV-associated diseases were similar between the two groups (*P*=0.317, *P*=0.802, *P*=0.534).

### CsA withdrawal and DLI

Of the 149 evaluable patients, 115 fulfilled the above criteria for CsA withdrawal from day +30. Of the 144 patients surviving day +60, 80 fulfilled the above criteria for prophylactic DLI (76 from day +60 and 4 from day +90), and 64 did not receive DLI. Twenty-one did not receive DLI due to the unavailability of donor lymphocytes. Other 43 with acute graft-versus-host disease (aGVHD) by day +30 or grade II/>II aGVHD by day +60 post-transplantation did not receive DLI, as a result of negative minimal residual disease (MRD) by day +90 (n=34), GVHD by day +90 (n=5) and death before day +90 (n=4).

### GVHD

Of the 149 evaluable patients, 34 developed grade I-IV aGVHD (grade I, n=8; grade II, n=14; grade III, n=8; grade IV, n=4) by day +30 post-transplantation and 2 died of aGVHD. Of the 115 patients withdrawing CsA, 34 developed aGVHD (grade I, n=16; grade II, n=13; grade III, n=5) by day +60 post-transplantation and one died from aGVHD. A total of 129 doses of DLI were administered in 80 patients, with a median of 2 (range, 1-4) doses per patient and a median dosage of 2.06 (range: 0.67-5.62) ×10^7^ CD3^+^ T cells/kg. Twenty-five developed (grade I, n=3; grade II, n=18; grade III, n=4) aGVHD, 52 developed chronic GVHD (cGVHD), and 1 died of aGVHD after DLI.

Overall, 89 patients developed aGVHD, and 77 of 138 surviving more than 100 days developed cGVHD. The 1-year cumulative incidences of overall and grade III-IV aGVHD were 62.1%±4.1% and 14.4%±2.9%, including aGVHD after DLI (overall: 31.6%±5.7%, grade III-IV: 3.9%±2.2%). The 2-year cumulative incidences of overall and extensive cGVHD were 61.4%±4.5% and 21.1%±3.7%. The 5-year mortality of GVHD was 14.1%±6.1%.

### Relapse

**Figure 1:**
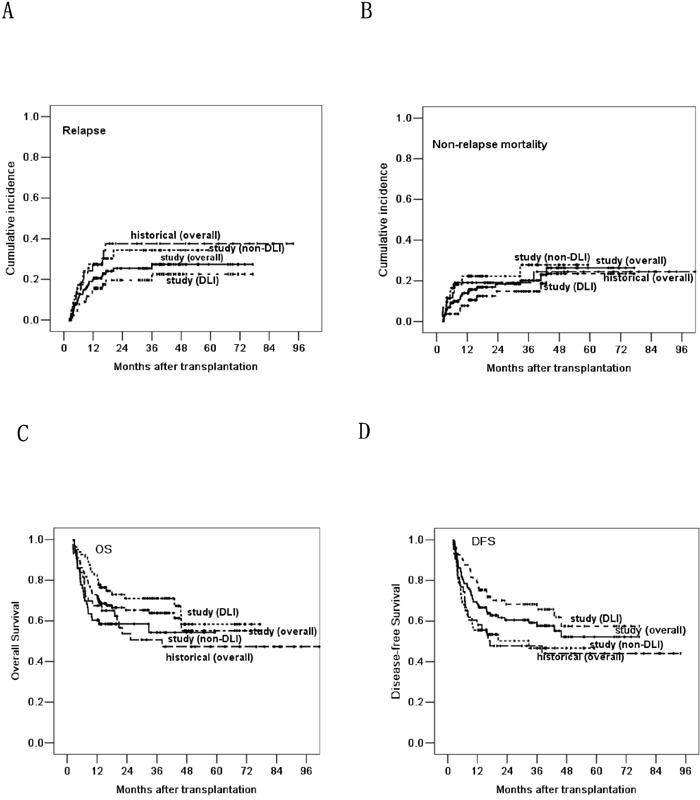
Based on the landmark analysis at 60 days, comparing the relapse rate A., non-relapse mortality B., overall survival C. and disease-free survival D. post-transplantation in the three groups of historical control, DLI and non-DLI

Thirty-two patients experienced leukemia relapse at a median time of 191 (range, 61 to 1079) days post-transplantation. Nine abandoned treatment and 23 received treatment, including 15 with chemotherapy and DLI, 5 with chemotherapy alone and 3 with chemotherapy and radiotherapy. Six achieved CR after treatment, and the others all died of disease progress or treatment-related complications. The 1-, 3- and 5-year cumulative incidence of leukemia relapse were 20.6%±3.5%, 27.3%±4.4% and 27.3%±4.4%. Fifteen of the 80 (18.8%) patients receiving DLI relapsed at a median time of 271 (range, 94 to 1079) days post-transplantation, and 17 of the 64 (26.6%) patients not receiving DLI relapsed at a median time of 165 (range, 61 to 611) days post-transplantation. The 5-year cumulative incidence of relapse in patients receiving DLI was 22.7%±5.5%, compared with 33.9%±7.2% in those not receiving DLI (*P*=0.048, Figure 1A). Univariate and multivariate analyses both revealed that DLI, cGVHD and lower bone marrow (BM) blasts on day 0 were associated with lower relapse (Table 3).

**Table 3:** Risk factors for leukemia relapse, OS and DFS

Risk factors	Relapse		OS		DFS	
	Univariate	Multivariate (HR)	Univariate	Multivariate (HR)	Univariate	Multivariate (HR)
male vs female	NS	NS	NS	NS	NS	NS
Age, < 29vs ≥ 29 years (median)	NS	NS	NS	NS	NS	NS
Disease category, AML/ALL/ABL	NS	NS	NS	NS	NS	NS
Genetic status, unfavorable vs other	NS	NS	NS	NS	NS	NS
Stage of treatment before HSCT, PIF vs other	NS	NS	NS	NS	NS	NS
Number of chemotherapy cycles pre-HSCT, < 4 vs ≥4 (median)	NS	NS	NS	NS	NS	NS
BM blasts before conditioning, <30.0% vs ≥30.0% (median)	NS	NS	NS	NS	NS	NS
Circulating blasts before conditioning, <11.0% vs ≥11.0% (median)	NS	NS	NS	NS	NS	NS
BM blasts on day 0, < 3.3% vs ≥3.3% (median)	P=0.001	P<0.001 (1.329) 95%CI: 1.197-1.475	P=0.008	P<0.001(1.236) 95%CI: 1.149-1.330	P=0.003	P<0.001(1.239) 95%CI: 1.151-1.333
Circulating blasts on day 0, none vs present	NS	NS	NS	NS	NS	NS
CD34^+^ counts in the graft, less than vs greater than or equal to median	NS	NS	NS	NS	NS	NS
Donor source, related vs unrelated donor	NS	NS	NS	NS	NS	NS
HLA typing, matched vs mismatched	NS	NS	NS	NS	NS	NS
No aGVHD vs I-II aGVHD vs III – IV aGVHD	NS	NS	NS	NS	NS	NS
No cGVHD vs limited cGVHD vs extensive cGVHD	P=0.026	P=0.036(0.474) 95%CI: 0.226-0.954	P=0.033	P=0.028 (0.573) 95%CI: 0.332-0.951	P=0.037	P=0.011(0.482) 95%CI: 0.268-0.866
No early CsA withdrawal vs early CsA withdrawal	NS	NS	NS	NS	NS	NS
No DLI vs DLI	P=0.033	P=0.048(0.495) 95%CI: 0.257-0.992	P=0.044	P=0.046 (0.623) 95%CI: 0.408-0.991	P=0.045	P=0.015(0.595) 95%CI: 0.399-0.888

OS=overall survival; DFS=disease-free survival;HR= hazard risk; vs=versus; AML= acute myelocytic leukemia; ALL= acute lymphoblastic leukemia; ABL= acute biphenotypic leukemia; HSCT= hematopoietic stem cell transplantation; PIF=primary induction failure; BM=bone marrow; HLA= human leukocyte antigen; aGVHD=acute graft-versus-host disease; cGVHD=chronic graft-versus-host disease; CsA=cyclosporine A; DLI= donor lymphocyte infusion; NS= not significant;CI=confidence interval.

### OS and DFS

With a median follow up of 421 (range, 0 to 2319) days post-transplantation, 92 patients survived and 61 died. Causes of death included leukemia relapse (n=26), infections (n=19, including 5 EBV-associated diseases), GVHD (n=9), diffuse alveolar hemorrhage (n=2), RRTs (n=2), cerebral hemorrhage (n=1), thrombotic microangiopathy (n=1) and secondary dyshematopoiesis (n=1). The 5-year NRM post-transplantation was 29.7%±5.3%, and it was similar between DLI and non-DLI groups (*P*=0.104, Figure 1B). The 5-year OS and DFS were 51.1%±5.7% and 49.2%±5.3%. The 5-year OS and DFS in patients receiving DLI were 58.1%±8.0% and 57.2%±7.5%, compared with 54.9%±7.0% and 47.3%±7.1% in those not receiving DLI (*P*=0.043, *P*=0.018, Figure 1C and 1D). Univariate and multivariate analyses both demonstrated that DLI and cGVHD were the protective factors for longer OS and DFS; higher BM blasts on day 0 was the only risk factor for longer OS and DFS (Table 3).

### Comparison with historical group

To maintain consistency of primary disease category and minimum follow-up (12 months) between the study and historical groups, we removed 6 patients with chronic myelogenous leukemia with blast crisis (CML-BC) from historical data, and re-analyzed historical data on December 31, 2009 [15]. A total of 48 refractory acute leukemia undergoing allo-HSCT but not receiving DLI from May 2001 to December 2008 were enrolled in the historical group (patient's characteristics as shown in Table 1). Except for one who died of infection and one who died of RRT on day +3 post-transplantation, 46 patients surviving more than 30 days were evaluable for engraftment, disease response and CsA withdrawal. Fourteen of the 48 (29.2%) patients experienced leukemia relapse at a median time of 209 (range, 35 to 511) days post-transplantation in the historical group. Two achieved CR after treatment, and the others all died of disease progress or treatment-related complications. With a median follow up of 566 (range, 3 to 3034) days post-transplantation, 21 patients survived and 27 died in the historical group. Causes of death included leukemia relapse (n=12), infections (n=6, including 1 EBV-associated diseases), GVHD (n=7), RRT (n=1) and second tumor (lung cancer, n=1).

To further evaluate the efficacy of DLI, we compared the outcomes of patients in the study group with our historical group. Based on the landmark analysis at 60 days, the outcome of patients receiving DLI in the study group was significantly improved in relapse rate, OS and DFS, when compared with the historical group (36.5%±8.4%, 47.3%±8.2% and 44.1%±8.0%) (*P*=0.045, *P*=0.031 and *P*=0.025, Figure 1). The relapse rate, OS and DFS were similar between historical and non-DLI groups (*P*=0.918, *P*=0.689 and *P*=0.877). The NRM was similar in the historical, DLI and non-DLI groups (*P*=0.261).

## DISCUSSION

For refractory leukemia undergoing allo-HSCT, the major obstacle is leukemia relapse [16, 17]. Reports in the literature indicate that the 5-year OS, DFS and relapse rate are estimated to range from 7% to 30%, 5% to 30%, and 44% to 63% in refractory leukemia post-transplantation [2, 17-19]. The intensity of the conditioning regimen has been shown to directly affect the relapse rate and DFS after allo-HSCT for refractory leukemia [20, 21]. Reduced-intensity conditioning (RIC) regimens have been advocated to reduce transplantation-associated toxicity in elderly or medically unfit patients; however, disappointing results have been reported with RIC transplantation in refractory leukemia [21, 22]. Intensified conditioning can reduce leukemia burden pre-transplantation and improve long-term survival for refractory leukemia [15, 20, 23]. The Flu/Ara-C regimen for salvage chemotherapy in refractory leukemia has a reported 40% to 50% probability of achieving CR [24, 25]. Based on these results, we used Flu/Ara-C regimen as salvage chemotherapy followed by a myeloablative regimen. In myeloablative regimen, TBI/CY regimen showed lower relapse and higher DFS than Bu/CY regimen for refractory acute leukemia [7, 26]. On account of the chemoresistance of drugs which patients with refractory acute leukemia were medicated with in previous treatment, VP-16 was applied less frequently and the possibility of resistance was comparatively lower. Therefore, we chose TBI/CY/VP16 as myeloablative regimen for refractory acute leukemia undergoing allo-HSCT. Our result showed that all 149 evaluable patients achieved CR at the time of neutrophil reconstitution, which confirmed the suitability of using Flu/Ara-C with TBI/CY/VP16 myeloablative regimen in refractory leukemia to decrease leukemia burden pre-transplantation and increase the CR rate post-transplantation. Meanwhile, it further verified our historical result that sequential intensified conditioning had an acceptable toxicity profile [15].

Immunosupressant withdrawal and DLI are generally used to induce GVL for refractory leukemia after allo-HSCT [27-30]. Our historical study adopted early rapid tapering of immunosuppressant to induce GVL, with 3-year relapse rate of 33.3% [15]. In this study, to enhance the GVL effect, DLI was conducted on the basis of early immunosuppressant withdrawal, reducing the relapse rate to 27.3%. Prophylactic DLI showed less relapse than no DLI. Multivariate analysis also revealed that DLI was the favorable factor for reducing relapse. These data suggested that interventions with prophylactic DLI post-transplantation could reduce relapse.

The two methods both face the risk of GVHD, especially in the early period post-transplantation. In our historical study, CsA was withdrawn by 20%/week in patients without aGVHD by day +30 post-transplantation, and the incidence of grade III-IV aGVHD was 11.5% [15]. In this study, to avoid an overwhelming GVHD, CsA was withdrawn by 10%/week in patients without aGVHD by day +30, and granulocyte colony-stimulating factor (G-CSF)-mobilized DLI was administrated in patients without grade II/>II aGVHD by day +60 or in those without GVHD but MRD positive by day +90. The results showed that the incidences of grade III-IV aGVHD after CsA withdrawal and DLI were 4.3% and 5.0%. The non-increased incidence of GVHD might be due to the following two reasons: the percentage of CSA withdrawal reduced from 20% per week in historical group to 10% in the study group; G-CSF-mobilized DLI had a comparative GVL effect but a lower morbidity and mortality of GVHD compared with steady DLI [29-31].

Intensified conditioning regimens might increase NRM, including RRTs and early infections [6, 9]. In this study, the mortality of RRTs was 1.3%, which had no significant difference compared with standard myeloablative conditioning [7, 32]. The incidence of infections in our cohort was similar to that in patients undergoing allo-HSCT with standard conditioning in a corresponding time period within 100 days post-transplantation, and was also similar to that reported in other literatures [33, 34]. Nonetheless, the 5-year NRM and mortality of GVHD post-transplantation were 29.7% and 14.1%. Therefore, decreasing NRM, especially the lethality of GVHD, is a worthy goal that merits further research.

Schmid and coworkers reported a very promising study that improved survival for refractory AML through a sequential regimen of Flu/Ara-c/amsacrine chemotherapy and reduced-intensity conditioning, along with immunosupressant withdrawal and prophylactic DLI, with 2-year OS and leukemia mortality of 40.0% and 39.3% [12]. In this study, we adopted the strategy of Flu/Ara-C salvage chemotherapy and TBI/CY/VP-16 myeloablative conditioning followed by early rapid tapering of immunosuppressant and prophylactic DLI, with 5-year OS and relapse rate of 51.1% and 27.3%. The favorable efficacy might be attributed to the two aspects: salvage chemotherapy and myeloablative conditioning decreased the leukemia burden at the time of transplantation; early tapering of immunosuppressant combined with DLI accelerated the GVL effect. Our strategy for refractory acute leukemia undergoing allo-HSCT was similar to that described by Schmid. Salvage chemotherapy was performed pre-conditioning, and the strategies of GVL induction, including immunosupressant withdrawal combined with prophylactic DLI, were conducted post-transplantation in the two strategies. However, our results were likely to be superior to theirs with respect to survival and relapse [12]. Along with the heterogeneity of patients included in our cohort, our protocol differed from theirs in terms of drugs used in conditioning, the interval between salvage chemotherapy and conditioning, intervention time for immunosupressant withdrawal and DLI, and other aspects. These might be the reasons why our result was superior. At the same time, there are some limitations of the study. For example, treatment modalities and doctor experience changed through years (historical group: from year 2001 to 2008; study group: from year 2009 to 2014). Since it is not a randomized study, patient selection bias and imbalanced features between groups could not be avoided. Well-designed prospective clinical trials are needed to establish better treatments for refractory acute leukemia.

In conclusion, prophylactic DLI achieves better outcomes than no DLI for refractory acute leukemia undergoing allo-HSCT with intensified conditioning. The strategy of sequential intensified conditioning followed by early immunosupressant withdrawal and DLI could reduce relapse of refractory acute leukemia after allo-HSCT.

## PATIENTS AND METHODS

### Patients and eligibility criteria

We conducted a prospective and non-randomized controlled study. Patients were included if they were refractory acute leukemia, not in CR pre-transplantation and age between 12 and 55 years. Refractory acute leukemia was defined according to the literature [35-37]. Exclusion criteria included: CML-BC, creatinine clearance <50 mL/min, bilirubin or transaminase level >2 times the upper limit of normal, cardiac shortening fraction <30% and pregnancy. The study performed in accordance with modified Helsinki Declaration, and the protocol was approved by respective ethical review boards before study initiation. All recipients, donors and/or guardians provided written informed consent.

### Conditioning regimen and strategies of inducing GVL

**Figure 2:**
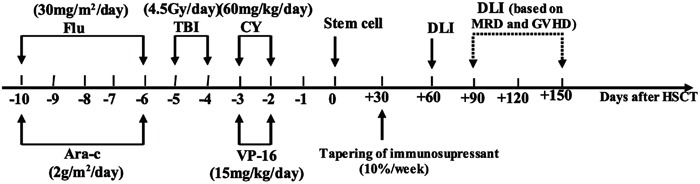
Protocol of sequential intensified conditioning followed by early tapering of immunosupressant and donor lymphocyte infusion for patients with refractory advanced acute leukemia undergoing allo-HSCT Flu, fludarabine; Ara-C, cytarabine; TBI, total body irradiation; CY, cyclophosphamide; VP-16, etoposide; DLI, donor lymphocyte infusion; MRD, minimal residual disease; GVHD, graft-versus-host disease; HSCT, hematopoietic stem cell transplantation.

**Figure 3:**
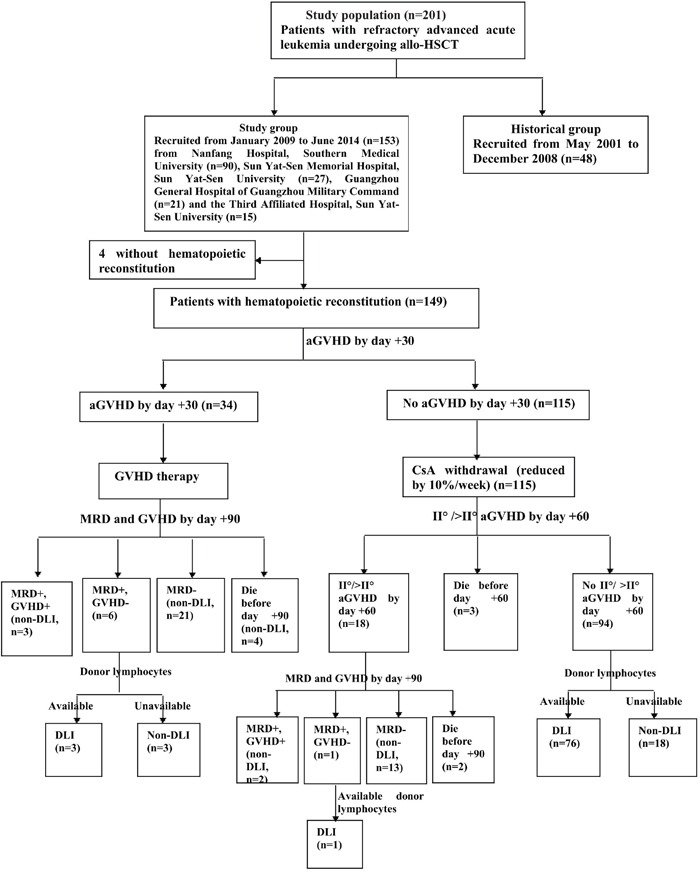
Strategies of inducing graft-versus-leukemia allo-HSCT, allogeneic hematopoietic stem cell transplantation; GVHD, graft-versus-host disease; aGVHD, acute GVHD; CsA, cyclosporine A; MRD, minimal residual disease; DLI, donor lymphocyte infusion.

The conditioning regimen was modified by increasing the dosage of VP-16 from 10 mg/kg/day to 15 mg/kg/day on the basis of our historical intensified conditioning (Figure 2) [15]. CsA withdrawal and DLI were conducted in patients who met the following criteria (Figure 3). CsA was withdrawn by 10%/week in patients without aGVHD by day +30, and ended in complete withdrawal by day +90 post-transplantation in patients without aGVHD. For patients without grade II/>II aGVHD by day +60 post-transplantation, G-CSF mobilized DLI was administered at a median dose of 1.0 (range 0.7-1.4) ×10^8^ mononuclear cells/kg if donor lymphocytes were available. DLI was given once to all patients regardless of MRD, and was then administered based on GVHD and MRD status. If patients were MRD negative, DLI was not given again; if patients were MRD positive and without GVHD, DLI was given monthly until GVHD occurred or MRD became negative or for a total of four times. For patients with aGVHD by day +30 or with grade II/>II aGVHD by day +60 post-transplantation, the application of DLI was based on the status of GVHD and MRD by day +90 and the availability of donor lymphocytes. If patients remained MRD+ and had no GVHD by day +90, DLI was administered according to the description above.

### Donor lymphocyte preparation

Donor lymphocytes were obtained from original donors. Collections were performed on the fifth day of G-CSF mobilization, and consecutive daily collections were performed until acquiring the target numbers of stem cells planned for transplantation and DLI. Donor lymphocytes was cryopreserved separately for DLI.

### GVHD prophylaxis and treatment

GVHD prophylaxis was described in our reports [15, 38]. CsA alone or CsA plus methotrexate (MTX) were administered in patients undergoing HLA-matched sibling donor transplants. CsA +MTX +antithymocyte globulin (ATG, Genzyme, Cambridge, MA) and/or mycophenolate were used in patients undergoing HLA-mismatched related and unrelated donor transplants. Methylprednisolone was used to treat aGVHD. CD25 monoclonal antibody (Novartis) alone or combined with ATG and other immunosupressant were used to treat glucocorticosteroid-resistant aGVHD. Corticosteroids and CsA in combination with various immunosupressant were used to treat cGVHD. Mesenchymal stem cells were also used to treat some patients with refractory aGVHD and cGVHD [39, 40].

### Infection prophylaxis

Acyclovir was given daily from the beginning of conditioning to engraftment, and was then administered daily for 7 days every 2 weeks until 1 year post-transplantation. Ganciclovir was given for 2 weeks pre-transplantation for prophylaxis of CMV infection, and was administered once again when CMV-emia occurred [4]. Preemptive therapy for EBV-emia was according to our previous description [38, 41]. Antifungal agents were administered 5 days pre-transplantation and continued for +30 to +90 days post-transplantation or disease control according to the history and state of IFD pre-transplantation [42].

### MRD monitoring

Two methods, including flow cytometry (FCM) and real-time quantitative polymerase chain reaction (RQ-PCR), were used for MRD detection. FCM positive was defined as >0.001% of cells with leukemia-associated aberrant immune phenotypes in BM samples post-transplantation. A total of 1 000 000 events were collected for analysis routinely. When cell numbers were limited, a minimal 750 000 events were collected. RQ-PCR was used for detection of leukemia-related genes including WT1, AML1/ETO, CBFβ/MYH11, MLL-PTD, MLL-AF9, MLL/AF4, BCR/ABL and E2A/PBX1 genes, defining the transcript level≥0.001% as PCR positive. MRD was monitored once a month in the first six months post-transplantation, once every two months until the 18th month, once every three months during the 19th to 36th month. If MRD was positive, it was monitored twice a month until MRD became negative. Subjects were scored as MRD positive if they had 2 consecutive positive results using FCM or PCR or were both FCM and PCR positive in a single sample.

### Evaluation points and definitions

Our study data was analyzed on June 30, 2015. The primary evaluation point was relapse, and secondary endpoints included engraftment, disease response, RRTs, infections, GVHD, NRM, OS and DFS. Leukemia relapse was defined as BM, extramedullary, or both by common morphological criteria. FCM and molecular data were not used to define relapse. Hematopoietic reconstitution, chimerism and disease response were defined according to described criteria [15]. RRTs were defined as toxicities directly due to the conditioning regimen, and were graded according to Bearman's criteria [43]; aGVHD and cGVHD were graded as described previously [44, 45]. Genetic subgroups were classified according to literature reported, including favorable, intermediate and unfavorable subgroups [46, 47]. NRM was defined as death from any cause other than relapse. EBV-associated disease was defined as infectious disease, rather than secondary tumor.

### Statistics

Comparisons of categorical variables were made by means of chi-squared tests or Fisher exact test. Differences between numerical variables were calculated by means of Mann-Whitney U-test. Incidence of time-dependent variables was estimated by Kaplan-Meier method. Univariate and multivariate Cox regression models were used to analyze risk factors for leukemia relapse and survival post-transplantation. Additionally, the landmark analysis at 60 days was used to compare the outcomes of the DLI and non-DLI in the study group with our historical group in terms of relapse rate, NRM, OS and DFS. Log-rank test was used for the analysis with *P* values reported as 2-sided and <0.05 as statistical significance.
